# Determination of Matrix Metalloproteinase 2 in Biological Samples Using a 3D Stochastic Microsensor Based on Graphene Oxide/AuNanoparticles/(Z)-N-(pyridin-4-yl-methyl) Octadec-9-enamide

**DOI:** 10.3390/ijms25126720

**Published:** 2024-06-18

**Authors:** Catalina Cioates Negut, Ruxandra-Maria Ilie-Mihai, Raluca-Ioana Stefan-van Staden

**Affiliations:** Laboratory of Electrochemistry and PATLAB, National Institute for Research and Development in Electrochemistry and Condensed Matter, 202 Splaiul Independentei Str., 060021 Bucharest, Romania; negutcatalina79@gmail.com

**Keywords:** three-dimensional stochastic microsensor, MMP-2, gastric cancer, biological samples

## Abstract

The levels of the MMPs in the biological samples of confirmed patients with gastric cancer are significantly elevated compared to those found in healthy people. Therefore, a novel 3D stochastic microsensor based on graphene oxide, modified with gold nanoparticles and (Z)-N-(pyridin-4-yl-methyl) octadec-9-enamide (namely N2-AuNP/GO), was designed for the determination of MMP-2 in biological samples, and validated for the screening tests of biological samples in order to be used for the early diagnosis of gastric cancer. The proposed sensor presents a low limit of quantification (1.00 × 10^−22^ g mL^−1^), high sensitivity (1.84 × 10^7^ s^−1^ g^−1^ mL), and a wide working concentration range (1.00 × 10^−22^–1.00 × 10^−7^ g mL^−1^). Recovery values higher than 99.15% were recorded for the assay of MMP-2 in whole blood, gastric tissue tumors, saliva, and urine samples.

## 1. Introduction

Early diagnosis of gastric cancer (GC) is needed to improve the state of health of the population by reducing the number of people diagnosed in later stages, as well as mortality due to gastric cancer. The primary methods employed for the clinical diagnosis of GC encompass endoscopic biopsy, imaging examinations, and blood testing [1,2]. Imaging tests, such as upper digestive tract X-rays and computed tomography, can aid in the diagnosis of GC. However, their use is limited due to the need for specialized equipment and skilled operators, making widespread implementation impractical.

Matrix metalloproteases (MMPs) are proteases encompassing gelatinases, stromelysins, collagenases, and membrane-type MMPs [3,4].

Upon recruitment, the immune cells secrete a plethora of mediators, including MMPs, into the tissue [5]. Collaborative activation, matrix remodeling, regulation of focal adhesion complexes, and protein shed at cellular junctions are all functions of multi-population MMPs. Cellular motility is affected because it reorganizes actin and causes disruptions in cellular connections. Extracellular matrix-bound cytokines and growth factors can also be activated by the matrix metalloproteinases. The growth factors and the MMPs that promote tumor cell motility and invasion are continuously produced by tumor cells and tumor-associated immune cells in the tumor microenvironment [6]. Through the promotion of mucosal damage and the facilitation of contact between the epithelium and bacteria, immune cells, and stroma cells, MMPs have the potential to further contribute to chronic inflammation [7]. 

During the process of tumor formation, it is possible that MMP-2 has a role in the control of tumor angiogenesis. This is accomplished by compromising the immune system, triggering the TGF-β signaling pathway, and expressing VEGF, as well as bFGF [8]. This involvement ultimately leads to the acceleration of tumor cell proliferation and the spread of tumor cells to distant locations. Because of this, MMPs have the potential to serve as a bridge among the tumor’s cells and the microenvironment of the tumor made up of GC cells.

The levels of MMPs in the biological samples from confirmed patients with GC are significantly elevated compared to those collected from healthy volunteers [9]. An increased expression of MMPs is strongly associated with the invasion and spread of tumors [10]. Hence, the precise and sensitive identification of MMPs is important in clinical cancer diagnosis and treatment. From this class of biomarkers, MMP-2 is a very important one for the diagnosis of cancer [11,12,13,14,15,16].

Early research has shown that human gastric cancer has a higher level of MMP-2 expression and activity compared to neighboring tissue. These findings have led researchers to propose that MMP-2 may be used as a prognostic marker for patients who have a low risk of overall survival [17].

This paper proposes the stochastic method as a screening method for biological samples and the identification and quantification of MMP-2 in biological samples. The stochastic sensors are highly selective and reliable screening tools used for both the qualitative and quantitative analysis of different biomarkers in any biological matrix: whole blood, gastric tissue tumor, saliva, and urine; their main advantage is that their response is not influenced by the complexity of the matrix from where the biomarker is determined.

Carbon-based materials (graphene oxide, GO) are excellent candidates for developing new types of microsensors [18,19] because they have exceptional properties, such as electrochemical activity, ease of surface functionalization, and the ability to ensure channel stability in the modifier used in the stochastic sensor design. Gold nanoparticles (AuNPs) offer several benefits, including unique physical and chemical properties that serve as excellent scaffolds for the development of new chemical and biological sensors [20], direct synthesis, stability, unique optoelectronic properties, a high surface-to-volume ratio with excellent biocompatibility when combined with suitable ligands, and the ability to both increase sensitivity and lower the detection limit of electrochemical sensors [21]. All these properties of AuNPs can be easily tuned by varying their size, shape, and surrounding chemical environment.

This work stands out from previous methods due to its innovative design of a 3D microsensor utilizing graphene oxide (GO) that has been modified with AuNP and (Z)-N-(pyridin-4-yl-methyl] octadec-9-enamide (N2) (an oleamide able to provide the requested channels for stochastic sensing). The inclusion of oleamide in the design is valuable because it improves the sensor’s electrochemical performance. The microsensor is specifically designed for the qualitative and quantitative detection of MMP-2 in whole blood, gastric tissue tumors, saliva, and urine samples, with the final purpose of being validated as new tool for the early diagnosis of gastric cancer.

## 2. Results and Discussion

### 2.1. Structural Characterization

**Figure 1:**
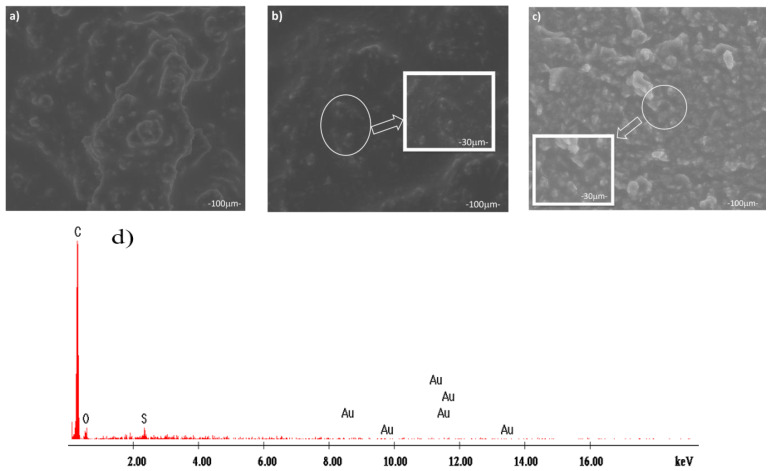
SEM images for (**a**) GO, (**b**) AuNP/GO, (**c**) N2-AuNP/GO pastes, (**d**) EDX spectrum for N2-AuNP/GO paste.

**Figure 2:**
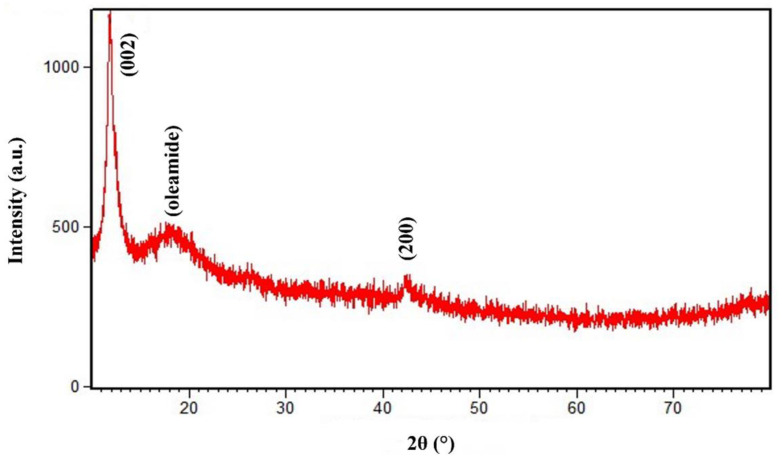
XRD diffractogram of the N2-AuNP/GO paste.

The SEM, EDX, and XRD are used to explore the morphological and structural properties of the samples, as well as to perform the semi-quantitative analysis of the materials that were analyzed. Figure 1 and Figure 2 provide the results of these investigations.

#### 2.1.1. SEM and EDX Analysis

In Figure 1a, the SEM image shows that the GO has a smooth surface. Furthermore, the arrows in Figure 1b,c indicate the presence of AuNP and oleamide particles, respectively. It can be seen that the gold nanoparticles shown in Figure 1b are distributed uniformly over the graphene surface. There are small clusters of AuNP that may be discovered in various locations. 

The oleamide nanoparticles can be observed on the surface of the graphene surface (Figure 1c). Simultaneous observations reveal the deposition of these nanoparticles not only on the graphene surface but also on top of the AuNP. Similar to the morphologies seen in previous Au/rGO-modified electrodes [22], a high-resolution scanning electron micrograph of the AuNP/GO and N2-AuNP/GO pastes (an enlarged partial view is provided in Figure 1b,c) further shows that the Au nanoparticles are firmly attached to the porous carbon surface.

Figure 1d illustrates the surface morphology of the materials that were investigated, as well as the semi-quantitative analysis performed by EDX. The analytical analysis revealed that all the samples shared the same elemental appearance, being primarily composed of carbon, oxygen, sulfur, and gold, with carbon accounting for around 93% of the mass. The morphological study reveals that the particles exhibit agglomerations or asymmetric forms.

#### 2.1.2. XRD Analysis

The XRD analysis was conducted to determine the degree of crystallinity shown by the nanoparticles in different amounts. Figure 2 displays the results. The XRD pattern shows two significant, low-intensity peaks. These peaks are at 10.3 degrees and 18 degrees, respectively, and correspond to GO and oleamide. AuNP may be responsible for a less intense third peak at 42.2 degrees. At 10.3° and 42.2°, the miller indices for the XRD diffraction peaks are 002 for GO and 200 for AuNP.

A comparable XRD pattern from a study reported by Krishnamurthy et al. [23] indicates that the synthesized AuNPs have a face-centered cubic (fcc) structure, as shown by the diffractogram and similar Bragg peaks. They found AuNP at 2θ = 44.3, which is very close to our findings. Furthermore, there are many additional studies [24,25] that have acquired XRD peaks with 2θ angles that are extremely close to the ones that we have found in our investigation. 

According to the research study by Kaneko et al. [26], in which they investigated the crystallization and crystal structure of the β phase of oleic acid, they conducted an XRD examination of their compound. This analysis revealed that they acquired reflections within 15 < 2θ < 30°. 

The polymorphism of oleamide was investigated in another study [27], which mostly utilized powder XRD as its method of investigation. At room temperature, the XRD diffractogram displays the D phase of oleamide. A Bragg angle with a value of around 23.5 degrees was found to correlate to a diffraction peak that was detected. It was found that there were parallels between these findings and our investigation. An 18° diffraction peak can be noticed in the XRD pattern that we obtained in our investigation. The two diffraction peaks derived from the pattern have comparable 2θ angles. As a result, we could assume that the diffraction peak we obtained can be attributed to the oleamide in our specific scenario. 

For the samples, there is a discernible widening of the diffraction peaks, which is indicative of the presence of crystallites that are smaller in size. The process of describing such a widening can be simplified by using the full width at half maximum (FWHM). If there is no lattice strain present in the specimen, we can determine the average size D of the crystallites (crystalline domains) using the Scherrer formula [28], as in Equation (1). This can be achieved by calculating the specimen broadening FWHM of the peaks in the observed pattern.

(1) $$D=\frac{k\lambda }{\beta \cos\left(\theta \right)}$$

where k is the dimensionless shape factor (for the fcc lattice, it is approximately 1), λ is the X-ray wavelength, which for our study is reported to be 1.5405, β is the line broadening at half the maximum intensity FWHM, and θ is the diffraction peak (Bragg angle). The calculated average crystallite sizes were 2.88 and 3.07 nm for the oleamide and AuNPs, respectively.

### 2.2. Response Characteristics of the 3D Stochastic Microsensor

**Figure 3:**
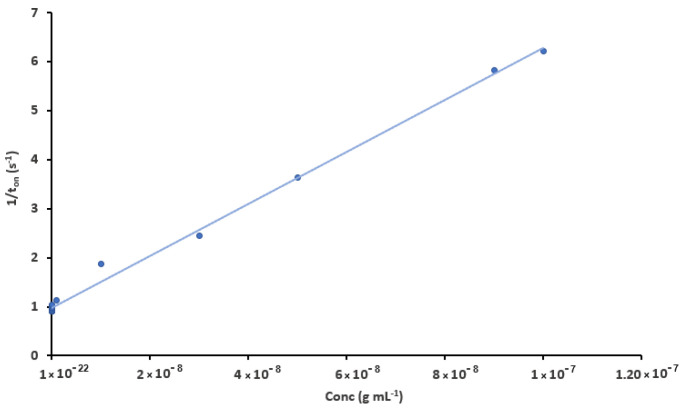
Calibration curve for the assay of MMP-2 using the 3D stochastic microsensor based on N2-AuNP/GO.

The response characteristics of the 3D stochastic microsensor were assessed using the chronoamperometry technique, by applying a voltage of 125 mV and keeping the temperature constant at 25 °C (Table 1). The calibration curve for the assay of MMP-2, using the 3D stochastic microsensor based on N2-AuNP/GO, can be seen in Figure 3.

The sensitivity of the sensor is high: 1.84 × 10^7^ s^− 1^ g^−1^ mL. A lower limit of quantification in the magnitude scale of zeptogram per milliliter (zg mL^−1^) was determined. The minimum detectable concentration of the analyte was determined based on the lowest concentration within the linear range of concentrations, as specified by the IUPAC recommendation [29]. The calculated detection limit for the analyte was considerably lower than the values published in the literature (Table 2). Compared to other methods listed in Table 2, the stochastic approach proposed in this study has multiple advantages. It is user-friendly, has lower sensitivity and limit of quantification, and allows for the direct measurement of biological samples without any pre-treatment. These characteristics make it a convenient choice for clinical applications.

### 2.3. Selectivity of the 3D Stochastic Microsensor

The t_off_ value, also known as the signature, of 1.1 s was obtained for MMP-2, as shown in Table 1. The selectivity assessment was performed by comparing it with the signatures recorded for other biomarkers, such as p53 (0.4 s), KRAS (1.8 s), MMP-3 (0.7 s), HER-3 (2.1 s), HER-4 (2.5 s), IL-2 (2.8 s), IDH-1 (3.5 s), CEA (3.2 s), and HRG-α (3.0 s) (Table 3). The results showed that the recorded signatures for the mentioned biomarkers are different from the one recorded for MMP-2, demonstrating that they do not interfere in the assay of MMP-2.

### 2.4. Validation of the Screening Method Used for Determination of MMP-2 in Biological Samples

**Figure 4:**
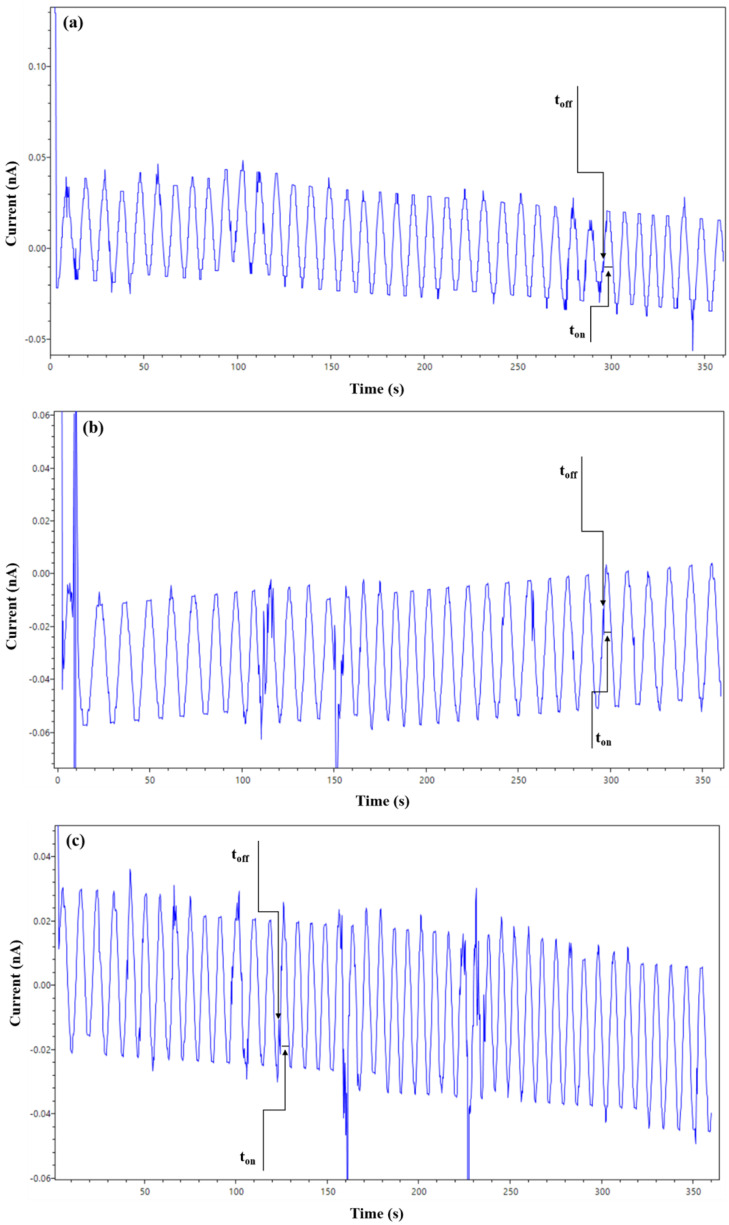

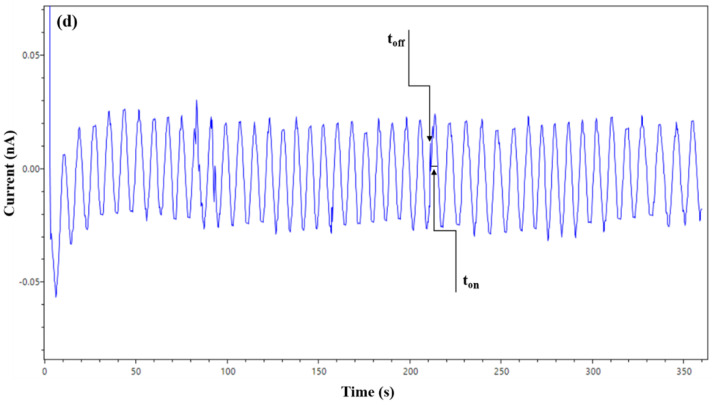
Stochastic diagrams recorded in (**a**) whole blood, (**b**) gastric tumor tissue, (**c**) saliva, and (**d**) urine samples, using the 3D stochastic microsensor based on N2-AuNP/GO.

The sensor was used to perform a qualitative and quantitative evaluation of MMP-2 in biological samples. Initially, the analyte was recognized in the diagrams based on its distinctive characteristics, as indicated in Table 1. The t_on_ value, which is used to calculate the concentration of each biomarker, was obtained by reading between two t_off_ values (Figure 4).

Table 4 presents the findings of the MMP-2 analysis in samples of whole blood, gastric tissue tumor, saliva, and urine taken from individuals with gastric cancer. The results obtained using the suggested stochastic microsensor showed a strong correlation with the traditional ELISA method during the screening of biological samples. This suggests that the stochastic microsensor is reliable, demonstrating the ability to determine the biomarker in different biological samples.

Very good correlations were obtained between the proposed method (stochastic method) and ELISA. These correlations were also proved by applying Student’s *t*-test at 99% confidence level, for each biological samples analyzed. At this confidence level, the tabulated value was 4.13. The values obtained for each biological sample were the following: 2.97 for the whole blood, 3.20 for the gastric tissue tumor, 2.20 for saliva, and 3.95 for urine. All the recorded values were lower than the tabulated value, proving that there is no significant difference between the results obtained using the two methods: the stochastic method and ELISA. Therefore, the stochastic method can be applied with high confidence for the assay of MMP-2 in the specified biological samples.

Recovery tests were conducted as second validation tests for the assay of MMP-2 in different biological samples, such as whole blood, gastric tissue tumors, saliva, and urine. Predefined levels of biomarkers were introduced into the biological sample and measured both before and after the addition of the predefined quantities. The data presented in Table 5 demonstrate that the biomarker shows accurate identification in biological samples, with recoveries higher than 99.15% and RSD values below 0.04.

The primary benefit of the assays of the biomarker is the reduced analysis time and lower cost: a 3D stochastic microsensor priced at under EUR 1 may deliver results within less than 10 min. This expedites the decision-making process for medical professionals in terms of diagnosis and therapy for their patients.

The second validation test proved once again the suitability of the proposed stochastic method and that of the newly designed 3D stochastic sensor for the screening of whole blood, gastric tissue tumor, urine, and saliva for MMP-2.

## 3. Materials and Methods

### 3.1. Instruments

For the morphology and quantitative analysis of the work pastes, a scanning electron microscope (SEM), Inspect S (FEI Europe B.V., Eindhoven, the Netherlands), was used. The Everhart-Thornley detector was utilized in high-vacuum mode, with a voltage of 30 kV and a magnification of 6000× *g* for the purpose of qualitative examination of the researched pastes. The SEM was equipped with an energy-dispersive X-ray detector (Inspect S + EDX, FEI, Eindhoven, the Netherlands) and operated in low-vacuum mode using the LFD detector. 

X-ray diffraction (XRD) data were collected using an X’Pert PRO MPD diffractometer (PANalytical, Almelo, the Netherlands) with Cu-Kα radiation in the 2θ range of 10–80°. 

The 3D system comprises three electrodes: a counter electrode made of platinum wire, a reference electrode made of Ag/AgCl (0.1 mol L^−1^ KCl), and a working electrode that was a 3D stochastic microsensor. Using a 3D Stratasys Objet 24 printer (Rehovot, Israel), we could create three-dimensional microtubes in the laboratory. 

The stochastic measurements were performed under normal environmental conditions using the EmSTAT Pico mini potentiostat (PalmSens BV, Houten, the Netherlands). The mini potentiostat was connected to a laptop, and the PSTrace program version 5.10 was used to control the experimental parameters and collect data.

All solutions were prepared using deionized water. To determine the pH values of the buffer solutions, we utilized a Mettler Toledo pH meter (Columbus, OH, UAS).

### 3.2. Reagents

Analytical grade materials such as graphene oxide (GO), gold nanoparticles (10 nm diameter, OD 1, suspension stabilized in 0.1 mM phosphate buffer solution (PBS), no reactants) (AuNPs), MMP-2, matrix metalloproteinases-3 (MMP-3), tumor protein P53 (p53), Kirsten rat sarcoma (KRAS), human epidermal growth factor receptor 3 (HER-3), human epidermal growth factor receptor 4 (HER-4), interleukin-2 (IL-2), isocitrate dehydrogenase 1 (IDH-1), carcinoembryonic antigen (CEA), heregulin-α (HRG-α) sodium phosphate monobasic monohydrate, sodium phosphate dibasic heptahydrate, hydrochloric acid, sodium hydroxide, and ethyl alcohol were used without any further purification and purchased from Sigma Aldrich (Milwaukee, USA). Fluka (Buchs, Switzerland) provided the paraffin oil. The compound (Z)-N-(pyridin-4-yl-methyl) octadec-9-enamide (N2) was prepared using the method as described elsewhere [56]. 

The MMP-2 solutions utilized for calibrating the sensor were prepared by the successive dilution approach, with concentrations ranging from 1.00 × 10^−7^ to 1.00 × 10^−22^ g mL^−1^ in a 0.1 mol L^−1^ phosphate buffer solution (PBS) with a pH of 7.40. PBS was obtained by mixing aqueous solutions of sodium phosphate monobasic monohydrate and sodium phosphate dibasic heptahydrate. Different proportions of the two solutions were combined until a pH of 7.4 was reached, and the pH was adjusted by adding hydrochloric acid or a sodium hydroxide solution with a concentration of 0.1 mol L^−1^.

### 3.3. Design of the 3D Stochastic Microsensor

**Scheme 1:**
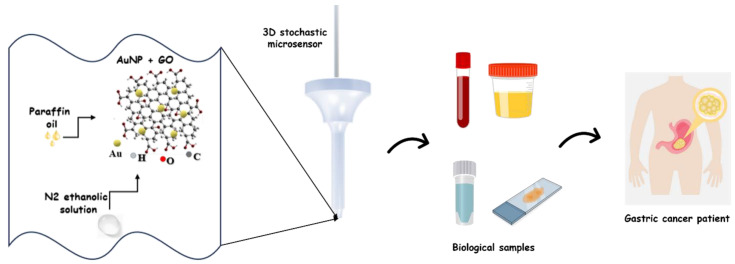
Schematic design of stochastic microsensor used as tool for screening tests.

A stochastic microsensor was designed using the following methodology: a homogenous paste was formed by mixing 100 mg of GO powder with 10 μL of AuNP dispersion (the optimal ratio for distinguishing the signal from the noise) and paraffin oil. To obtain the modified paste, a 100 μL solution of oleamide (N2, 1.00 × 10^−3^ mol L^−1^, prepared in ethyl alcohol) was added as modifier by carefully dropping it into the mixture (Scheme 1). A silver wire was used to establish a connection between the paste and the external circuit. The microsensors underwent a cleaning operation using distilled water, followed by a drying step after each measurement. The microsensors were kept in a cool environment and protected from light while not being in use.

### 3.4. Procedure

For this study, the stochastic method was used, which is capable of performing both the quantitative and qualitative analysis of one or more analytes present in any type of sample. The stochastic sensor is based on the principle of channel/pore conductivity [57], which occurs in two phases. The analyte’s distinguishing characteristic is the specific pore blocking-up time (t_off_) (analyte signature), which demonstrates the individuality of each analyte. The value of the pattern recognition phase in qualitative analysis is directly proportional to the duration of the analyte-specific phase. The period in which the analyte makes contact with the wall of the pore, achieving a state of equilibrium, is referred to as the equilibrium time, and is measured as t_on_. We use this measurement to quantitatively determine the analyte’s concentration. The calibration equation, expressed as 1/t_on_ = a + b × Conc_biomarker_, was derived using linear regression. We applied a constant potential of 125 mV (optimized value) in all measurements. The analysis of solutions containing variable concentrations of biomarkers was performed within a time interval of 360 s, while the analysis of biological samples was performed within 600 s.

### 3.5. Samples

Forty biological samples, including whole blood, gastric tissue tumor, saliva, and urine, were obtained from the Emergency Clinical Hospital of Targu-Mures County and the Clinical Hospital of Targu-Mures County. Their Ethics Committees granted permission for this research, with the numbers 32647/14.12.2018 and 3206/28.02.2019, respectively. Samples were procured after receiving written consent from all patients. No pre-treatment of the samples was conducted before their analysis.

## 4. Conclusions

The novel 3D stochastic microsensor based on graphene oxide, modified with gold nanoparticles and (Z)-N-(pyridin-4-yl-methyl) octadec-9-enamide (namely N2-AuNP/GO), was successfully used for the assay of MMP-2 in whole blood, gastric tumor tissue, saliva, and urine. The validation of the sensors and the screening method proved that they are suitable to be reliably used for the assay of MMP-2 in biological samples. The feature of the proposed method and sensor is their utilization for early diagnosis of gastric cancer, aiming to reduce both mortality due to gastric cancer and the incidence of late-stage diagnoses of gastric cancer.

## Figures and Tables

**Table 1:** The response characteristics of the 3D stochastic microsensor used for the assay of MMP-2.

Analyte	Linear Concentration Range (g mL^−1^)	Calibration Equation and Correlation Coefficient (r) *	t_off_ (s)	Sensitivity (s^−1^ g^−1^ mL)	LOQ (g mL^−1^)	LOD (g mL^−1^)
MMP-2	1.00 × 10^−22^–1.00 × 10^−7^	1/t_on_ = 0.16 + 1.84 × 10^7^ × C; r = 0.9999	1.1	1.84 × 10^7^	1.00 × 10^−22^	2.59 × 10^−24^

* C—concentration = g mL^-1^; t_on_ = s; LOQ—limit of quantification; LOD—limit of detection.

**Table 2:** Comparison of various methods used for MMP-2 detection.

Sensing Method/Probe	Method	Detection Range (g mL^−1^)	Detection Limit (g mL^−1^)	Ref.
Apt-9-Apt-2-PDANS	Fluorescence	6.40 × 10^−11^–1.60 × 10^−8^	2.56 × 10^−11^	[30]
DNA-peptide-Cy3	Fluorescence	2.73 × 10^−10^–8.62 × 10^−8^	2.40 × 10^−10^	[31]
UCP−peptide−CNP FRET	Fluorescence	1.44 × 10^−8^–1.44 × 10^−7^	3.61 × 10^−9^	[32]
PMPD-Peptide-FITC nanoprobe	Fluorescence	7.20 × 10^−9^−1.44 × 10^−6^	2.30 × 10^−9^	[33]
Peptide microarray	Fluorescence	2.50 × 10^−11^–5.00 × 10^−7^	1.40 × 10^−11^	[34]
Peptide-coated UCNPs	Fluorescence	1.00 × 10^−9^–2.00 × 10^−7^	2.20 × 10^−9^	[35]
MBs-SA/biotin-KKGRVGLPGC-DNA	Fluorescence	7.18 × 10^−14^–7.18 × 10^−10^	4.31 × 10^−14^	[36]
GO-Pep-FITC- FRET	FRET	1.00 × 10^−8^–1.50 × 10^−7^	2.50 × 10^−9^	[37]
Eu^3+−^BCTOTpeptide-AMC	FRET	5.00 × 10^−10^–4.00 × 10^−8^	1.25 × 10^−8^	[38]
GO-peptide-QDs nanoprobes	FRET	-	7.00 × 10^−8^	[39]
PSi microsensor	Optical refractivity	1.70 × 10^−10^–2.64 × 10^−7^	1.70 × 10^−10^	[40]
Au/MCH/peptide1-Ir1; Au/MCH/peptide2-Ru1	ECL	1.00 × 10^−8^–3.00 × 10^−7^	5.00 × 10^−9^	[41]
nanopillar chip/CO-nanotags-PEG-peptide	SERS	5.00 × 10^−8^–2.00 × 10^−5^	5.00 × 10^−11^	[42]
Ni-NTA MB- iTRAQ-coded peptide	UPLC-MS/MS	0.20 × 10^−9^–1.00 × 10^−7^	6.40 × 10^−11^	[43]
AuNPs/MCH-gelatin	Colorimetry	2.00 × 10^−8^–6.00 × 10^−7^	2.00 × 10^−8^	[44]
CS-AuNPs-Pb/peptide/AuNPs/polyaniline gel/GCE	SWV	1.00 × 10^−12^–1.00 × 10^−6^	4.00 × 10^−13^	[45]
Au/PAMAM/GM-Fc/PPD/GL-AQ/AuNPs/CGI-MB	SWV	1.00 × 10^−8^–1.00	6.20 × 10^−16^	[46]
MCH/peptide/Zr(IV)/CPAD/Fc	SWV	1.00 × 10^−12^–1.00 × 10^−9^	2.70 × 10^−13^	[47]
Au@Pt nanorods	Electrochemical	5.00 × 10^−10^–1.00 × 10^−7^	1.80 × 10^−10^	[48]
GO–peptide fluorescence sensor	Potentiometry	3.50 × 10^−9^–3.00 × 10^−8^	2.60 × 10^−9^	[49]
peptide/AuNPs@HRP@ZIF-8	DPV	1.00 × 10^−14^–1.00 × 10^−8^	5.33 × 10^−15^	[50]
MSFs/[Ru(NH_3_)_6_]Cl_3_	DPV	1.00 × 10^−11^–2.00 × 10^−8^	9.8 × 10^−10^	[51]
PSC-peptide-AuNPs-DNA_1_	DPV	5.00 × 10^−13^–5.00 × 10^−8^	1.50 × 10^−13^	[52]
FC-modified peptide ligand	DPV	1.00 × 10^−9^–2.00 × 10^−7^	3.00 × 10^−10^	[53]
MCH/Fc-peptide/depAu/GCE	DPV	1.00 × 10^−13^–2.00 × 10^−8^	3.00 × 10^−14^	[54]
Dual-channel detection using Au-QDs core-satellite nanoprobes	ASV	1.00 × 10^−12^–5.00 × 10^−10^	6.30 × 10^−13^	[55]
Stochastic sensor	Stochastic	1.00 × 10^−22^–1.00 × 10^−7^	2.59 × 10^−24^	This paper

AMC: 7-amino-4-methylcoumarin; AQ: antraquinone-2-carboxylicacid; ASV: anodic stripping voltammetry; AuNPs: gold nanoparticles; AuNPs@HRP@ZIF-8: zeolite imidazolate frameworks-8 loaded with horseradish peroxidase (HRP) and gold nanoparticles; Au-QD: gold-quantum dot; BCTOT: 1,10-bis(5′-chlorosulfo-thiophene-2′-yl)-4,4,5,5,6,6,7,7-octafluorodecane1,3,8,10-tetraone; C: chronoamperometry; CNPs: carbon nanoparticles; CPAD: 4-cyano-4-(phenylcarbonothioylthio)pentanoic acid; CS: chitosan; Cy3: cyanine fluorophore; DPV: differential pulse voltammetry; ECL: electrochemiluminescence; Fc: ferrocenecarboxylic acid; FC: ferrocenyl acetic acid; FITC: fluorescein isothiocyanate; FRET: fluorescence resonance energy transfer; GO: graphene oxide; Ir1: iridium(III) complex (dfppy)_2_Ir(dcbpy)PF_6_; iTRAQ: isobaric tags for relative and absolute quantification conjugates; MB: methylene blue; MBs-SA: streptavidin-coated magnetic beads; MCH: 6-mercapto-1-hexanol; MSFs: mesoporous silica films; NiNTA MBs: nickel-nitrilotriacetic acid-modified magnetic beads; PAMAM: polyamidoamine; PDANS: polydopamine nanosphere; PEG: polyethylene glycol; Pep-FITC: fluorescein isothiocyanate-labeled peptide; PMPD: poly(m-phenylenediamine); PPD: p-phenylenediamine PSC: polystyrene microsphere; Psi: porous silicon; Ru1: Ru complex (Ru(bpy)_2_(mcbpy-O-Su-ester)(PF_6_)_2_); SERS: surface-enhanced Raman scattering; SWV: square wave voltammetry; UCNPs: upconversion nanoparticles; UCPs: upconversion phosphors; UPLC-MS/MS: ultrahigh-performance liquid chromatography–tandem mass spectrometry.

**Table 3:** The selectivity of the microsensor.

t_off_ (s), Signature									
MMP-2	p53	KRAS	MMP-3	HER-3	HER-4	IL-2	IDH-1	CEA	HRG-α
1.1	0.4	1.8	0.7	2.1	2.5	2.8	3.5	3.2	3.0

**Table 4:** Determination of MMP-2 in biological samples (N = 10).

Analyte	Biological Samples	Sample Number									
		1	2	3	4	5	6	7	8	9	10
MMP-2 (ng mL^−1^) Stochastic method	Whole blood	3.77 ± 0.01	6.48 ± 0.02	12.89 ± 0.01	27.40 ± 0.01	27.58 ± 0.03	81.81 ± 0.01	18.33 ± 0.02	32.98 ± 0.01	40.59 ± 0.02	29.97 ± 0.01
	Gastric tissue tumor	19.76 ± 0.02	1.58 ± 0.01	59.14 ± 0.02	23.22 ± 0.02	2.70 ± 0.01	36.28 ± 0.02	5.44 ± 0.01	12.05 ± 0.02	12.89 ± 0.03	81.99 ± 0.02
	Saliva	29.99 ± 0.01	3.21 ± 0.03	172.50 ± 0.01	127.16 ± 0.02	27.40 ± 0.02	14.79 ± 0.01	15.85 ± 0.03	81.81 ± 0.01	23.17 ± 0.01	7.61 ± 0.03
	Urine	40.64 ± 0.01	59.14 ± 0.03	14.79 ± 0.01	21.59 ± 0.03	27.13 ± 0.03	83.63 ± 0.02	18.33 ± 0.02	17.24 ± 0.01	59.14 ± 0.02	36.28 ± 0.01
MMP-2 (ng mL^−1^) ELISA	Whole blood	3.21 ± 0.14	6.18 ± 0.28	13.00 ± 0.26	25.97 ± 0.32	28.04 ± 0.18	81.05 ± 0.22	17.97 ± 0.75	31.76 ± 0.33	41.23 ± 0.29	30.00 ± 0.25
	Gastric tissue tumor	19.20 ± 0.21	1.22 ± 0.76	61.05 ± 0.20	23.11 ± 0.39	3.00 ± 0.29	36.00 ± 0.30	5.10 ± 0.11	12.40 ± 0.23	11.90 ± 0.29	82.00 ± 0.14
	Saliva	30.00 ± 0.40	3.00 ± 0.29	170.12 ± 0.43	127.00 ± 0.22	26.96 ± 0.60	13.20 ± 0.89	15.12 ± 0.56	80.99 ± 0.20	21.98 ± 0.38	7.00 ± 0.45
	Urine	40.12 ± 0.24	60.22 ± 0.54	15.15 ± 0.26	21.00 ± 0.14	27.20 ± 0.58	84.77 ± 0.43	18.00 ± 0.24	16.80 ± 0.20	60.00 ± 0.43	36.20 ± 0.25

**Table 5:** Recovery tests of MMP-2 in biological samples using the 3D stochastic microsensors (N = 10).

Biological Samples	Whole Blood	Gastric Tissue Tumor	Saliva	Urine
%, MMP-2 recovery	99.98 ± 0.02	99.15 ± 0.04	99.76 ± 0.02	99.37 ± 0.01

## Data Availability

The original contributions presented in the study are included in the article, further inquiries can be directed to the corresponding authors.
